# Oxidation of P700 Ensures Robust Photosynthesis

**DOI:** 10.3389/fpls.2018.01617

**Published:** 2018-11-06

**Authors:** Ginga Shimakawa, Chikahiro Miyake

**Affiliations:** ^1^Department of Biological and Environmental Science, Faculty of Agriculture, Graduate School of Agricultural Science, Kobe University, Kobe, Japan; ^2^Core Research for Environmental Science and Technology, Japan Science and Technology Agency, Tokyo, Japan

**Keywords:** P700 oxidation, photosystem I, photoinhibition, reactive oxygen species, photosynthesis

## Abstract

In the light, photosynthetic cells can potentially suffer from oxidative damage derived from reactive oxygen species. Nevertheless, a variety of oxygenic photoautotrophs, including cyanobacteria, algae, and plants, manage their photosynthetic systems successfully. In the present article, we review previous research on how these photoautotrophs safely utilize light energy for photosynthesis without photo-oxidative damage to photosystem I (PSI). The reaction center chlorophyll of PSI, P700, is kept in an oxidized state in response to excess light, under high light and low CO_2_ conditions, to tune the light utilization and dissipate the excess photo-excitation energy in PSI. Oxidation of P700 is co-operatively regulated by a number of molecular mechanisms on both the electron donor and acceptor sides of PSI. The strategies to keep P700 oxidized are diverse among a variety of photoautotrophs, which are evolutionarily optimized for their ecological niche.

## Introduction

Nothing ventured, nothing gained. In oxygenic photosynthesis, CO_2_ is converted into sugar using H_2_O and light energy. Additionally, O_2_ is released from oxygenic photosynthesis as a by-product. Thus, oxygenic photoautotrophs (i.e., cyanobacteria, algae, and plants) support life on Earth with only sun light energy. However, this process is not risk-free, and every photoautotroph is constantly exposed to potential photo-oxidative damage.

Oxygenic photosynthesis is achieved through the assimilation of CO_2_ from the atmosphere in the Calvin-Benson cycle (dark reaction) and the utilization of light for the production of nicotinamide adenine dinucleotide phosphate (NADPH) and adenosine triphosphate (ATP) to meet the demand for CO_2_ assimilation in the photosynthetic electron transport system (light reaction). Light energy from the sun is absorbed by pigments, such as chlorophyll (Chl), in light-harvesting complexes (LHC) around two photosystems (PSII and PSI) on the thylakoid membrane, and excites the reaction center Chls (P680 and P700 in PSII and PSI, respectively) to drive charge separation and photosynthetic linear electron flow from PSII to PSI via plastoquinone (PQ) pool, cytochrome (Cyt) *b*_6_/*f* complex, and plastocyanin (PC) or Cyt *c*_6_ (Barber and Andersson, 1994). On the electron donor side of PSII, photo-oxidized P680 (P680^+^) oxidizes H_2_O with O_2_ evolved with the help of the oxygen-evolving complex (Nathan and Wolfgang, 2015). On the electron acceptor side of PSI, NADP^+^ is reduced to NADPH with electrons from P700 via ferredoxin (Fd) and Fd-NADP^+^ reductase (FNR). Photo-oxidized P700 (P700^+^) is reduced with electrons from PSII via the Cyt *b*_6_/*f* complex and PC (or Cyt *c*_6_) in photosynthetic linear electron flow (Jensen et al., 2007). The proton gradient across the thylakoid membrane (ΔpH), which is the motive force of the chloroplast ATP synthase (ATPase), is established at both the steps of H_2_O oxidation on the luminal side of PSII and electron transport in the Q-cycle of the Cyt*b*_6_*f* complex (Cramer et al., 2006). NADPH and ATP produced in the photosynthetic electron transport system are utilized for driving CO_2_ assimilation in the Calvin-Benson cycle (Calvin and Benson, 1948) and are required to be in concordance. However, dynamic natural environmental variations can easily unbalance the production and utilization of NADPH and ATP, resulting in photo-oxidative damage to photosynthetic cells.

Absorption of light energy exceeding the demand for photosynthetic CO_2_ assimilation can cause inactivation of these photosystems. This light-dependent inactivation of photosynthesis was first observed in the green alga *Chlorella* sp. more than 50 years ago and was termed photoinhibition by Kok (1956). Based on the characterization of photoinhibition using isolated thylakoid membranes, it has been separately recognized as deriving from the inactivation of PSII or PSI (Satoh, 1970). Photoinhibition of PSII leads to light-dependent degradation of the reaction center subunit (D1 protein), which is observed *in vivo* under various stress conditions, such as high light, high temperature, and drought. Photoinhibited PSII can rapidly recover by replacing the degraded D1 protein with a newly synthesized D1 protein in several hours. The processes that lead to the photo-oxidative damage of PSII are still under debate, although numerous studies have provided extensive and remarkable insights into the mechanisms of PSII photoinhibition (Krause et al., 1985; Aro et al., 1993; Sundby et al., 1993; Andersson and Barber, 1996; Neidhardt et al., 1998; Melis, 1999; Allakhverdiev and Murata, 2004; Hakala et al., 2005; Murata et al., 2007; Tyystjärvi, 2008; Fischer et al., 2013; Pospíšil, 2016; Jimbo et al., 2018).

In this review, we concentrate on the photoinhibition of PSI, which depends on both O_2_ and electrons produced by PSII (Satoh, 1970). Compared with studies on PSII photoinhibition, those on PSI photoinhibition are relatively rare, since it hardly occurs *in vivo*, even if oxygenic photoautotrophs are exposed to a stress treatment with excess light (e.g., continuous light illumination with high light) (Critchley, 1981; Powles and Björkman, 1982; Havaux and Eyletters, 1991). Previous studies, using isolated thylakoid membranes and chloroplasts, have suggested that PSI photoinhibition is derived from a dysfunction in the [4Fe–4S] clusters on the acceptor side of PSI (i.e., F_X_, F_A_, and F_B_), caused by reactive oxygen species (ROS) (Satoh, 1970; Inoue et al., 1986). PSI photoinhibition was first observed *in vivo* in the intact leaves of the chilling-sensitive plant *Cucumis sativus* under chilling stress (Terashima et al., 1994). The characterization of PSI photoinhibition under such conditions corroborated previous findings from *in vitro* studies and worked toward establishing the present theory of the mechanisms of PSI photoinhibition (Sonoike, 2011).

On the electron acceptor side of PSI, excess photo-excitation energy can reduce O_2_, generating ROS, including superoxide anion radical (O_2_^-^), hydrogen peroxide (H_2_O_2_), and hydroxyl radical (⋅OH) (Mehler, 1951; Asada, 2006; Rutherford et al., 2012). Owing to their high reactivity, ROS can immediately inactivate PSI (Sonoike, 1996; Sonoike et al., 1997). In comparison with PSII, the damaged PSI takes a long time (days or weeks) to completely recover (Kudoh and Sonoike, 2002; Zivcak et al., 2015b). Therefore, PSI photoinhibition is a lethal event for oxygenic photoautotrophs. That is why PSI photoinhibition hardly occurs *in vivo* except for under specific conditions, such as chilling. The mechanisms of prevention of PSI photoinhibition had remained unknown for long.

## P700 Oxidation and its Physiological Significance

The generation of ROS in PSI should be strictly suppressed for the purpose of preventing PSI photoinhibition *in vivo*. Based on the simple concept of oxygenic photosynthesis, the electron acceptor side of PSI is expected to be over-reduced when the Calvin-Benson cycle cannot follow the production of NADPH in the photosynthetic electron transport system. Nevertheless, PSI is always kept in an oxidized state in response to situations where the Calvin-Benson cycle is suppressed, which has been observed using *in vivo* spectroscopic measurement techniques for P700^+^ (Foyer et al., 1990; Harbinson and Hedley, 1993; Klughammer and Schreiber, 1994; Golding and Johnson, 2003; Miyake et al., 2005). The universal physiological response of oxygenic photoautotrophs is termed “P700 oxidation” and refers to the increase in the ratio of P700^+^ to the total amount of photo-oxidizable P700. In comparison with P700, P700^+^ cannot drive its photo-oxidation/reduction cycle but directly dissipate the photo-excitation energy as heat (Nuijs et al., 1986; Trissl, 1997; Bukhov and Carpentier, 2003). Therefore, P700 oxidation is expected to be directly linked to the quenching of excess light energy in PSI.

Recently, the impact of P700 oxidation on the alleviation of PSI photoinhibition has been demonstrated by a method to easily and selectively induce PSI photoinhibition in intact plant leaves at room temperature (Sejima et al., 2014). In the method named “repetitive short-pulse (SP) illumination (rSP illumination),” SP light (e.g., 300-1000 ms, 2000-20,000 μmol photons m^-2^ s^-1^) is repetitively applied to plant leaves (e.g., every 10 s) under darkness. This experimental procedure is similar to continuously shooting the plants with a camera with a strobe light at night and can be defined as a severe form of artificial fluctuating light. This is different to illumination with continuous light; during rSP illumination, PSI is inactivated significantly faster than PSII in intact plant leaves, depending on the intensity and length of the SP light (Sejima et al., 2014; Zivcak et al., 2015b). Eliminating or limiting O_2_ suppress the inactivation of PSI, indicating that PSI photoinhibition during rSP illumination is caused by ROS (Sejima et al., 2014). This observation corresponds to results of previous studies on chilling-induced PSI photoinhibition (Sonoike, 1996; Sonoike et al., 1997). Therefore, rSP illumination is a useful tool to induce PSI photoinhibition *in vivo* (Zivcak et al., 2015a,b; Kono and Terashima, 2016; Takagi et al., 2017b; Mikko and Steffen, 2018).

**FIGURE 1:**
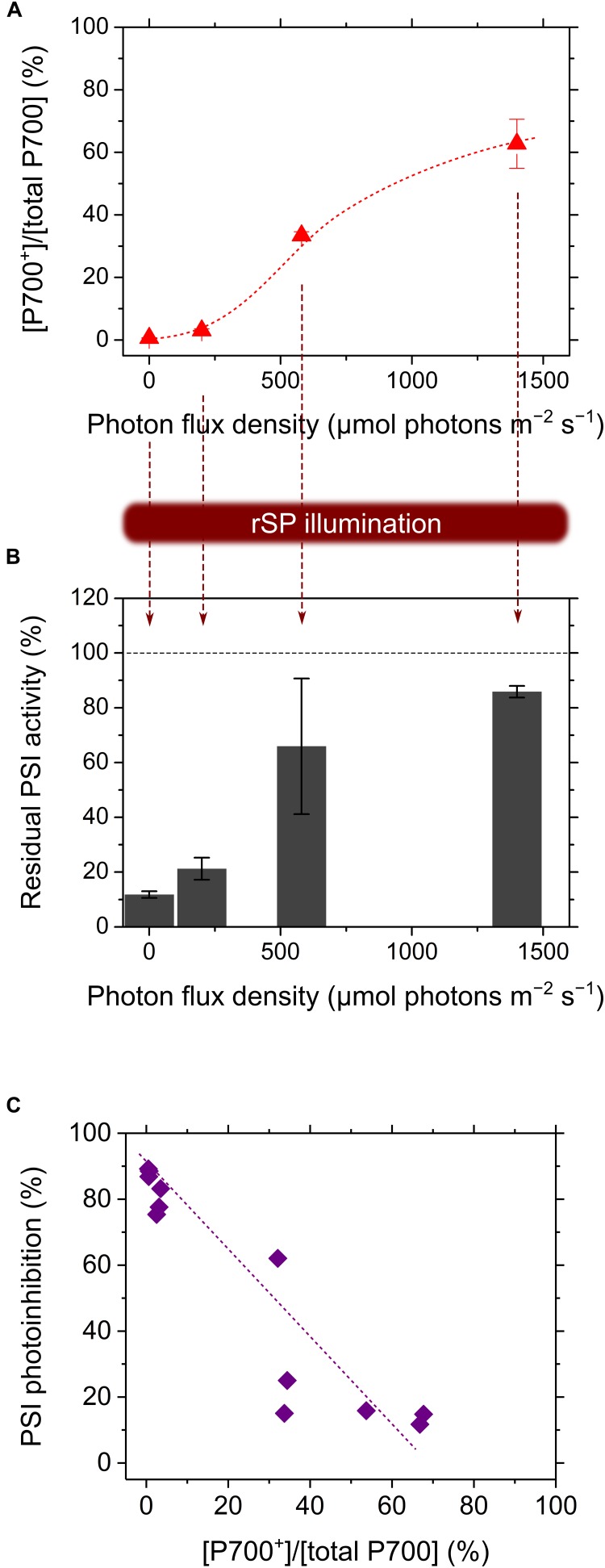
Relationship of P700 oxidation with the alleviation of the photo-oxidative damage in PSI during exposure to repetitive short-pulse (rSP) illumination. The sunflower plant leaves were exposed to rSP illumination (20,000 μmol photons m^-2^ s^-1^, 300 ms, every 10 s) in different light intensities producing different P700 oxidation levels **(A)**. Residual photochemical activity of PSI was evaluated as the residual total photo-oxidizable P700 after rSP illumination for 4 h **(B)**, and the decrease in the total photo-oxidizable P700 was plotted against the P700 oxidation levels **(C)**. Data were from Sejima et al. (2014).

**FIGURE 2:**
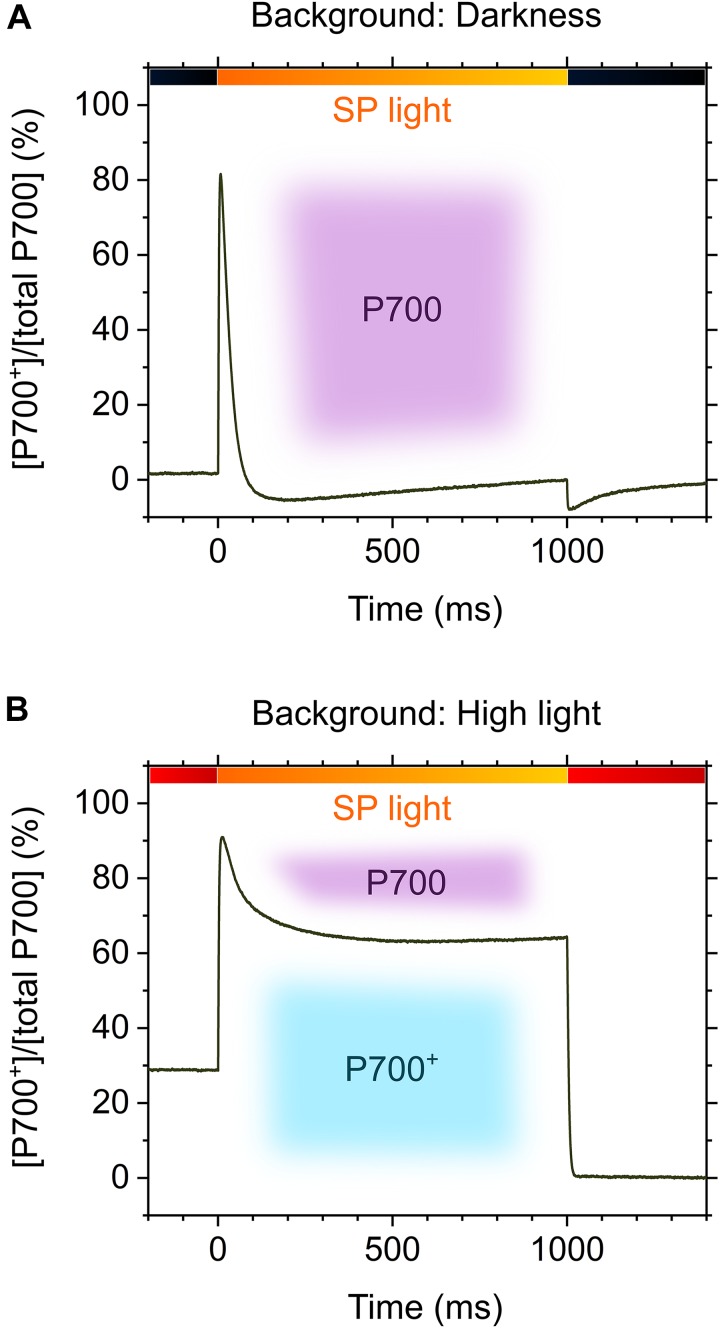
Kinetic model of oxidized P700 during exposure to short pulse (SP) illumination (2000-20,000 μmol photons m^-2^ s^-1^, 1 s) in the leaves of angiosperms in the absence **(A)** and presence **(B)** of background high light. Black bars, darkness; red bars, high light; orange bars, SP light. Purple and sky-blue shadings indicate P700 in the reduced and oxidized forms, respectively.

Sejima et al. (2014) have applied rSP illumination to sunflower leaves under constant actinic light at different intensities, producing different P700 oxidation levels (Figures 1A,B), resulting in a linear relationship of P700 oxidation with the alleviation of PSI photoinhibition (Figure 1C) (Sejima et al., 2014). The effects of P700 oxidation on the protection of PSI against photoinhibition is also evidenced by the kinetics of P700^+^ in response to SP light during rSP illumination. In the intact leaves of angiosperms, P700 is excited by SP light and is kept in a reduced state during exposure to SP light (Figure 2A), which suggests that electron transport in PSI is limited on the acceptor side, but not on the donor side, during exposure to SP light. Contrarily, in the presence of a continuously high intensity background light, P700 is kept in an oxidized state during exposure to SP light (Figure 2B), which is due to a change in the limitation step of the electron transport system in PSI from acceptor to donor sides by P700 oxidation system (described in the next chapter). Furthermore, the addition of a far-red light in the background during rSP illumination can also stimulate P700 oxidation in the SP light to suppress PSI photoinhibition (Kono et al., 2017), which might suggest that shaded plants in an understory can efficiently keep P700 oxidized during natural “sunflecks.”

**FIGURE 3:**
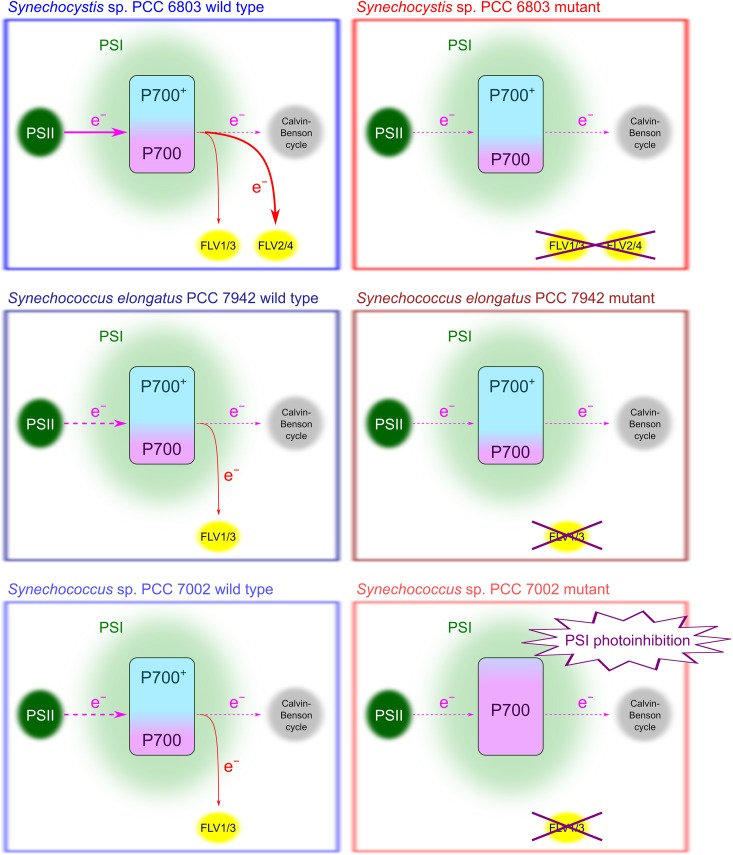
Relationship of P700 oxidation with the alleviation of the photo-oxidative damage in PSI during exposure to constant light. Both wild types and flavodiiron protein (FLV)-deficient mutants of the three cyanobacteria species grown under high-[CO_2_] conditions show the different responses of the photosynthetic electron transport system to the suppression of the Calvin-Benson cycle under CO_2_ limitation: *Synechocystis* sp. PCC 6803 expresses FLV2/4 to mediate O_2_-dependent alternative electron transport but can keep P700 oxidize even without the FLV-mediated alternative electron sink; *Synechococcus elongatus* PCC 7942 suppresses photosynthetic linear electron flow and oxidizes P700 regardless of the existence of FLV; and *Synechococcus* sp. PCC 7002 suppresses photosynthetic linear electron flow and oxidizes P700 with the FLV-mediated alternative electron sink. Among these cyanobacterial cells, PSI photoinhibition is observed only in the mutant of *Synechococcus* sp. PCC 7002 that cannot keep P700 oxidized (Shimakawa et al., 2016b). All arrows indicate electron transport direction, and suppressed electron transport is represented by dashed arrows.

The impact of P700 oxidation on the alleviation of PSI photoinhibition has been observed not only during rSP illumination but also continuous light. In cyanobacteria, the progenitor of oxygenic photosynthesis, P700, is kept in an oxidized state in response to a suppression of photosynthesis under CO_2_ limitation, similarly to that in intact plant leaves (Badger and Schreiber, 1993). Shimakawa et al. (2016b) validated the common response of the redox state of PSI to CO_2_ limitation in three different cyanobacteria species, *Synechocystis* sp. PCC 6803, *Synechococcus elongatus* PCC 7942, and *Synechococcus* sp. PCC 7002 (Figure 3). Among the mutants deficient in flavodiiron protein (FLV) in each species, only the mutant of *Synechococcus* sp. PCC 7002 cannot keep P700 oxidized under CO_2_ limitation and suffers from PSI photoinhibition. These facts led us to suggest that the fate of PSI is determined by whether P700 can be kept in an oxidized state under excess light conditions. Furthermore, the inactivation of PSI in the mutant of *Synechococcus* sp. PCC 7002 has been observed even in the range of photon flux density between 200 and 300 μmol photons m^-2^ s^-1^ (Shimakawa et al., 2016b), which indicates that PSI has the potential to generate ROS and suffer from PSI photoinhibition even under constant light 10 times less intense than sunlight, if the light exceeds the demand of the electron sinks, such as the Calvin-Benson cycle. Overall, without P700 oxidation, oxygenic photoautotrophs would easily suffer from PSI photoinhibition under natural environmental variation. The diverse strategies to keep P700 oxidized in these cyanobacteria species are further discussed at the section “*Flavodiiron Protein (FLV)*” in the chapter “*Regulatory Mechanisms to Keep P700 in an Oxidized State, P700 Oxidation System.*”

Besides being a quencher of light energy to suppress the generation of ROS in PSI, P700^+^ is assumed to play other important roles. For example, the charge recombination of P700^+^ with the electron acceptors in PSI (e.g., F_X_) can occur in the range of the μs- or ms-order half times (Semenov et al., 2000). That is, P700^+^ can act as an electron sink to oxidize [4Fe–4S] clusters on the acceptor side of PSI, which might suppress the generation of ⋅OH (Sonoike, 1996). Additionally, P700^+^ possibly thermally dissipate excess light energy not only in PSI but also around PSII via energy transfer mechanisms, including state transition and/or spillover (Ueno et al., 2018; Yokono and Akimoto, 2018), which ultimately, also has the potential to alleviate PSII photoinhibition.

## Regulatory Mechanisms to Keep P700 in an Oxidized State, “P700 Oxidation System”

**FIGURE 4:**
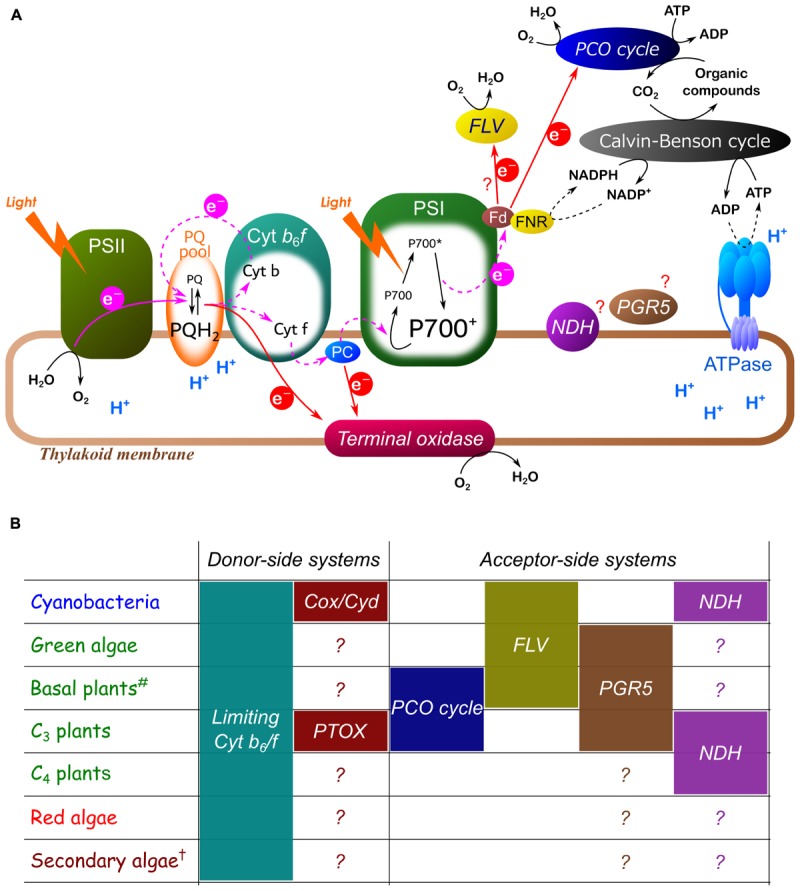
Hypothetical model for P700 oxidation system **(A)** and summary table of the diversity observed in a variety of oxygenic photoautotrophs **(B)**. Pink and red arrows represent photosynthetic linear and alternative electron transport respectively. Black lines represent various reactions, including oxidation, reduction, and phosphorylation. Dashed lines indicate suppressed reactions. Each P700 oxidation system is shown in italics. Limiting Cyt *b*_6_/*f* indicates the suppression of electron transport in Cyt *b*_6_/*f* by lumen acidification and/or reduction-induced suppression of electron flow (RISE). Cox, *aa*_3_-type Cyt *c* oxidase; Cyd, Cyt *bd* quinol oxidase; PTOX, plastid terminal oxidase; PCO, photorespiratory carbon oxidation; FLV, flavodiiron protein; PGR5, proton gradient regulation 5; NDH, chloroplast NADPH dehydrogenase. The mechanisms inducing P700 oxidation by PGR5 and NDH are still controversial. ^#^Basal plants mean liverworts, mosses, ferns, and gymnosperms. ^†^Secondary algae contain many different groups, including Chlorarachniophyta, Euglenophyta, Cryptophyta, Haptophyta, Heterokontophyta, and Dinophyta, and we note that the molecular mechanisms for P700 oxidation are still poorly understood in all these groups. The Euglenoid *E. gracilis* possibly utilize photorespiration (Yokota and Kitaoka, 1987; Shimakawa et al., 2017b). The dinoflagellate *Symbiodinium* sp. exceptionally have analogous genes for FLV and present a large O_2_-dependent alternative electron sink (Roberty et al., 2014). Micro and macro algae categorized into Cryptophyta, Haptophyta, and Heterokontophyta show P700 oxidation in response to short-pulse illumination with different dependencies on O_2_ (Shimakawa et al., 2018a). Generally, Glaucophyta is characterized by the same definition (Archaeplastida) as Chlorophyta (green algae) and Rhodophyta (red algae), but in this study we do not review P700 oxidation system in Glaucophyta because of scant existing literature on the subject. But recently, the glaucophyte *Cyanophora paradoxa* has been reported to develop cyanobacteria-like regulatory mechanisms of the photosynthetic electron transport system (Misumi and Sonoike, 2017).

P700 oxidation is strictly regulated by diverse molecular mechanisms (collectively termed P700 oxidation system) in oxygenic photoautotrophs (Figure 4). Importantly, the redox state of P700 depends on both the electron donor and acceptor sides of PSI. There are various regulatory mechanisms functioning on both sides of PSI. P700 oxidation is commonly observed in oxygenic photoautotrophs in response to excess light conditions, and the strategies to keep P700 in an oxidized state are diverse. Many diverse ways to oxidize P700 have been recognized already in the photosynthetic prokaryote cyanobacteria (Shimakawa et al., 2016b; Figure 3), which have supposedly developed and changed during the evolutionary history of oxygenic photoautotrophs.

### Limiting Electron Transport in Cyt *b_6_/f*

On the donor side of PSI, the suppression of electron transport into PSI causes P700 oxidation. Photosynthetic linear electron flow has been recognized as being limited to the oxidation of reduced PQ (i.e., plastoquinol, PQH_2_) in Cyt *b*_6_/*f* without any specific regulatory mechanisms at moderate lumen pH values (6.5-7.5). This is based on the understanding that the oxidation of PQH_2_ is the slowest step in the photosynthetic electron transport system, and that the amount of Cyt *b*_6_/*f* is normally smaller than those of PSII and PSI in plant leaves (Stiehl and Witt, 1969; Anderson, 1992; Schöttler and Tóth, 2014), which is supported by a linear relationship between Q_A_ reduction and P700 oxidation (Shimakawa and Miyake, 2018a). The limitation of electron transport in Cyt *b*_6_/*f* is likely to be a common strategy to keep P700 in an oxidized state in oxygenic photoautotrophs, considering the following regulatory mechanisms.

Electron transport in Cyt *b*_6_/*f* is modulated by a regulatory mechanism, which is believed to be strongly associated with lumen acidification (Nishio and Whitmarsh, 1993). Lumen acidification is linked to photosynthetic linear electron flow since ΔpH is provided by both the oxidation of H_2_O at the luminal side of PSII and the Q-cycle in Cyt *b*_6_/*f* (Schreiber and Neubauer, 1990; Avenson et al., 2005). Additionally, cyclic electron flow around PSI (CEF) can also promote the formation of ΔpH (Nandha et al., 2007). Contrarily, lumen acidification is stimulated by narrowing the proton efflux in ATPase and ion channels on the thylakoid membrane (Takizawa et al., 2008; Armbruster et al., 2014), leading to P700 oxidation for the alleviation of PSI photoinhibition (Takagi et al., 2017a). These processes are often considered as photosynthetic control (Foyer et al., 1990; Schöttler and Tóth, 2014).

Electron transport in Cyt *b*_6_/*f* can be suppressed in response to not only ΔpH but also the reduction in the PQ pool, which has been recently proposed as reduction-induced suppression of electron flow (RISE) in cyanobacteria (Shaku et al., 2016; Shimakawa et al., 2018b). In the Q-cycle (Mitchell, 1966), PQH_2_ donates one electron to Cyt *f* through a [2Fe–2S] cluster at Q_p_ (or Q_o_) site in Cyt *b*_6_/*f* and the other electron to a PQ at Q_n_ (or Q_i_) site in Cyt *b*_6_/*f*. The PQ at the Q_n_ site is reduced with the second electron originating from PSII. Therefore, a shortage of PQ can inhibit the operation of the Q-cycle and suppress electron transport in Cyt *b*_6_/*f*. Unfortunately, the molecular mechanisms of RISE remain poorly understood and have not yet been properly characterized. However, suppression of electron transport in Cyt *b*_6_/*f* is relieved by alternative electron flow mediated by FLV (Shaku et al., 2016) and exogenously added H_2_O_2_ (Shimakawa et al., 2018b). Here, we use the term “alternative electron flow” as the electron transport uncoupled with photosynthesis (i.e., the Calvin-Benson cycle). In cyanobacteria, FLV mediates the electron transport to O_2_ in PSI (Helman et al., 2003) and exogenously added H_2_O_2_ stimulates the electron transport via peroxidase utilizing the electrons in PSI (Miyake et al., 1991), both of which theoretically consume the electrons on the acceptor side of PSI but produce ΔpH (Schreiber and Neubauer, 1990). These facts, the electron transport in Cyt *b*_6_/*f* is modulated by not only lumen acidification (Trubitsin et al., 2003) but also another mechanism sensitive to the reduction of the photosynthetic electron transport system in cyanobacteria. The effect of RISE on P700 oxidation is possibly observed also in C_3_ plants (Takagi et al., 2016a; Shimakawa and Miyake, 2018a), but further research is required.

### Thylakoid Terminal Oxidases

Terminal oxidases on the thylakoid membrane, including plastid terminal oxidase (PTOX) and cyanobacterial respiratory terminal oxidases (Cox, *aa*_3_-type Cyt *c* oxidase; and Cyd, Cyt *bd* quinol oxidase), are also defined as a P700 oxidation system, on the electron donor side of PSI. These oxidases are localized on the thylakoid membrane and donate electrons from the interchain of the photosynthetic electron transport system: i.e., PQH_2_, PC, and Cyt *c*_6_ to O_2_ (Pils et al., 1997; Joët et al., 2002). Thus, the electrons on the donor side of PSI can be leaked to O_2_, which has an impact on the redox state of P700 during the transition from dark to light (Bolychevtseva et al., 2015; Feilke et al., 2016).

Particularly in cyanobacteria, the respiratory electron transport system shares the same PQ pool with the photosynthetic electron transport system and can have a large effect on photosynthesis, compared with photosynthetic eukaryotes (Shimakawa et al., 2014; Misumi et al., 2016). Recently, in the cyanobacterium *Synechocystis* sp. PCC 6803, both Cox and Cyd have been found to contribute to P700 oxidation and the alleviation of PSI photoinhibition during rSP illumination (Shimakawa and Miyake, 2018b). Unfortunately, the electron transport capacities of these terminal oxidases are yet to be quantitatively determined to be established as a suitable alternative electron sink for P700 oxidation in the cyanobacterial cells *in vivo* (Schuurmans et al., 2015). Oxidation of the PQ pool by both Cox and Cyd in the darkness is likely to help P700 oxidation to be induced in response to a light illumination. Further, these respiratory terminal oxidases can pump H^+^ into the luminal side of the thylakoid membrane (Paumann et al., 2005), possibly stimulating the limitation of electron transport in Cyt *b*_6_/*f* by photosynthetic control.

The chloroplast of photosynthetic eukaryotes harbors PTOX, a non-heme diiron carboxylate protein showing sequence similarity to the mitochondrial alternative oxidase, which binds strongly to the stromal side of the thylakoid membrane and functions in the process of chlororespiration to oxidize the PQ pool (McDonald et al., 2011; Johnson and Stepien, 2016). It has been reported that the alternative electron flux through PTOX is not functional for the protection of PSII and PSI against photoinhibition at the steady-state photosynthesis (Rosso et al., 2006). At least in the mature tissues of plants the amount of PTOX is 100 times smaller than that of PSII (Lennon et al., 2003). On the other hand, the recombinant PTOX protein of rice harbors the enough O_2_ reducing activity comparable to photosynthetic linear electron transport (*k*_cat_, >20 s^-1^; Yu et al., 2014). Additionally, the heterologous expression of *Chlamydomonas reinhardtii* PTOX in the tobacco leads to P700 oxidation (Feilke et al., 2016). These facts indicate that PTOX has the potential to contribute to P700 oxidation for the alleviation of PSI photoinhibition in photosynthetic eukaryotes in the situations where the relative amount of PTOX to photosystems increases (Rumeau et al., 2007).

### Photorespiration

On the electron acceptor side of PSI, an alternative electron sink, uncoupled with photosynthesis, supports P700 oxidation by relieving limitation of PSI on the acceptor side. Photorespiration is initiated by the oxygenation reaction of ribulose 1,5-bisphosphate (RuBP) carboxylase/oxygenase (so-called Rubisco) with 3-phosphoglycerate and 2-phosphoglycolate produced from RuBP and CO_2_ (Berry et al., 1978; Ogren, 1984). In the processes for regeneration of 3-phosphoglycerate from 2-phosphoglycolate in photorespiratory carbon oxidation (PCO) cycle, both reduced Fd and ATP are required. Additionally, both RuBP and CO_2_ regenerated by photorespiration are utilized again for CO_2_ assimilation in the Calvin-Benson cycle. That is, photorespiration can function as an O_2_-dependent alternative electron sink to dissipate excess light energy (Powles et al., 1979; Kozaki and Takeba, 1996; Takahashi et al., 2007). Indeed, photorespiration functions as the largest alternative electron flow to O_2_ (Badger et al., 2000; Ruuska et al., 2000; Driever and Baker, 2011; Sejima et al., 2016), and is responsible for P700 oxidation and the protection of PSI against photoinhibition in C_3_ plant leaves (Wiese et al., 1998; Takagi et al., 2016a; Wada et al., 2018).

In contrast to C_3_ plant leaves, photorespiration does not function as an alternative electron sink for P700 oxidation in cyanobacteria and algae. Though the genes for PCO cycle enzymes are commonly conserved in oxygenic photoautotrophs, a variety of algae, including cyanobacteria, green algae, and diatoms, show little O_2_-dependent electron sink capacity derived from photorespiration, even under CO_2_ limitation (Bidwell and McLachlan, 1985; Weger et al., 1989; Hayashi et al., 2014; Shimakawa et al., 2015, 2016a, 2017b). In aquatic environments the air-equilibrated O_2_ concentration is approximately 250 μM at 25C° and the diffusion coefficient of O_2_ decreases to approximately 0.01% of that in the atmosphere, which probably makes it difficult to utilize photorespiration, considering that the oxygenation reaction of RuBP catalyzed by Rubisco has significantly low affinity for O_2_. Despite of the large varieties, the *K*_m_ values have been recently reported to be in the range between 100 and 1600 μM at 25C° in diverse oxygenic photoautotrophs except for the Rubisco of Archaea (Tcherkez, 2016; Orr et al., 2016). As mutants of cyanobacteria and algae deficient in the genes for the PCO cycle are impaired in their growth, photorespiration is assumed to play other important roles, rather than acting as an alternative electron sink in these photoautotrophs (Eisenhut et al., 2006; Rademacher et al., 2016). O_2_-dependent electron transport activity with low affinity is observed in *Euglena gracilis* (Euglenophyta) (Shimakawa et al., 2017b); the secondary alga harboring chloroplasts is believed to be derived from green algae (Falkowski et al., 2004), which may suggest that *E. gracilis* uniquely utilizes photorespiration as an electron sink (Yokota and Kitaoka, 1987). Interestingly, photorespiration-derived electron sink comparable to the Calvin-Benson cycle is observed in liverworts, ferns, gymnosperms, and angiosperms except for in C_4_ plants (Hanawa et al., 2017). These data indicate that photorespiration had started to function as a large alternative electron sink since oxygenic photoautotrophs were first exposed to high partial pressures of O_2_ in the atmosphere.

### Flavodiiron Protein (FLV)

The protein family of FLV (or FDP) is defined based on two domains: a diiron center and a flavin mononucleotide-binding, and reduces O_2_ and NO directly into H_2_O and N_2_O using coenzymes such as rubredoxin and F_420_ (Romão et al., 2016). In addition, FLV in oxygenic photoautotrophs harbors a unique domain, similar to a flavin:NAD(P)H oxidoreductase, and therefore has been characterized by an ability to catalyze the reduction of O_2_ directly to H_2_O, with NAD(P)H as the electron donor (Vicente et al., 2002). The physiological function of FLV has been well characterized in the cyanobacterium *Synechocystis* sp. PCC 6803 in the pioneering work of Helman et al. (2003). The authors indicate that FLV mediates an O_2_-dependent alternative electron flow, probably on the acceptor side of PSI, and supports P700 oxidation. The electron sink capacity of FLV-mediated electron transport is evidenced by both the measurement of ^18^O_2_ photoreduction (Helman et al., 2003; Allahverdiyeva et al., 2013; Burlacot et al., 2018) and the simultaneous evaluation of O_2_ evolution with Chl fluorescence (Shimakawa et al., 2015). On the contrary, the physiological electron donor for FLV is still unknown. Some recombinant FLV proteins of the cyanobacterium *Synechocystis* sp. PCC 6803 show NAD(P)H-dependent O_2_ reduction into H_2_O, but the reduction rates are more than 100 times smaller than those of anaerobic bacteria (Vicente et al., 2002; Di Matteo et al., 2008; Shimakawa et al., 2015). In addition, it has been suggested that FLV interacts with Fd in *Synechocystis* sp. PCC 6803 (Hanke et al., 2011). The molecular mechanisms of FLV still await biochemical validation *in vitro*.

The impact of FLV on P700 oxidation is diversified already in the photosynthetic prokaryote cyanobacteria. Three cyanobacterial species show the different responses of the regulation of photosynthetic electron transport to CO_2_ limitation: *Synechocystis* sp. PCC 6803 expresses FLV2/4 to induce the large alternative electron flux to O_2_ uncoupled with photosynthesis; *Synechococcus elongatus* PCC 7942 suppresses the electron transport in Cyt *b*_6_/*f*; and *Synechococcus* sp. PCC 7002 keeps the electron transport capacity with the alternative electron transport to O_2_ through FLV1/3 dependent on the CO_2_ concentration of the growth conditions (Figure 3; Shimakawa et al., 2016a,b). Among these three cyanobacteria species, only in *Synechococcus* sp. PCC 7002 the FLV-knockout mutant suffers from PSI photoinhibition due to the inability to keep P700 oxidized in the situation where photosynthesis is suppressed under CO_2_ limitation (Figure 3; Shimakawa et al., 2016b). That is, FLV is the dominant regulator for the redox state of P700 in this species. On the other word, the other two species can keep P700 oxidized even in the absence of FLV by relying on the other regulatory mechanisms (Figure 3). Unfortunately, it is still unclear what regulatory mechanisms complement the capacity for P700 oxidation in the FLV-knockout mutants of these two species. Shaku et al. (2016) suggests that the limitation of electron transport in Cyt *b*_6_/*f* by RISE has the large impact on P700 oxidation in *Synechococcus elongatus* PCC 7942. Additionally, from the fact that the genes for Cyd are missed in the genome of *Synechococcus* sp. PCC 7002, different from the other two species (Shimakawa et al., 2016b), the terminal oxidase is suggested to be one possibility to complement P700 oxidation in the absence of FLV in *Synechocystis* sp. PCC 6803 and *Synechococcus elongatus* PCC 7942 (Shimakawa and Miyake, 2018b).

Among the oxygenic photoautotrophs, the genes for FLV are conserved in Cyanophyta (cyanobacteria), Chlorophyta (green algae), Bryophyta (liverworts and mosses), Pteridophyta (ferns), gymnosperms, and limited secondary algae (e.g., *Symbiodinium* sp.), in which P700 is rapidly oxidized in response to light exposure in the presence of O_2_ to alleviate PSI photoinhibition (Allahverdiyeva et al., 2013; Shirao et al., 2013; Roberty et al., 2014; Gerotto et al., 2016; Shimakawa et al., 2016b, 2017a; Chaux et al., 2017; Ilík et al., 2017; Noridomi et al., 2017; Takagi et al., 2017b). Interestingly, angiosperms have lost FLV at the genetic level (Allahverdiyeva et al., 2015; Yamamoto et al., 2016; Alboresi et al., 2018). Most importantly, P700 oxidation, but not FLV, is essential for oxygenic photoautotrophs to protect PSI against photoinhibition (Shimakawa et al., 2016b). In other words, FLV is not required if P700 can be kept oxidized in excess light conditions without it. Indeed, wild-type plant leaves of angiosperms can rapidly induce P700 oxidation except for during artificial severe stress conditions such as rSP illumination (Takagi et al., 2017b; Shimakawa and Miyake, 2018a). Additionally, most of the red algae (Rhodophyta) and secondary algae that have red plastid, including Cryptophyta, Haptophyta, and Heterokontophyta (diatoms, brown algae, etc.), can rapidly induce P700 oxidation in response to excess light and alleviate PSI photoinhibition during rSP illumination in the absence of FLV (Shimakawa et al., 2018a), implying that FLV is not completely required for oxygenic photoautotrophs already at the time that red algae had birthed. It would not be unexpected for angiosperms to have lost FLV during their evolutionary history. In these oxygenic photoautotrophs without FLV, P700 oxidation should be relying mainly on other regulatory mechanisms as mentioned in this chapter (e.g. limiting electron transport in Cyt *b*_6_/*f*).

The requirements of FLV are diverse in a variety of oxygenic photoautotrophs, likely depending also upon their ecological niche. The liverwort *M. polymorpha* preferably utilizes the alternative electron sink of FLV, but not photorespiration, when it is submerged (Shimakawa et al., 2017a). Taking the high affinity of the reaction with O_2_ (*K*_m_, a few or less μM) (Vicente et al., 2002; Shimakawa et al., 2015) into consideration, FLV probably provides better benefits than photorespiration under water. Additionally, the exposure to a far-red light in the terrestrial fields possibly affect the strategies to utilize FLV in a variety of basal land plants (Kono et al., 2017).

### Proton Gradient Regulation 5 (PGR5)

A number of studies have reported that the 10 kDa thylakoid membrane-associated protein, called PGR5, is essential to keep P700 in an oxidized state in green algae and land plants. A lack of PGR5 creates a profound limitation in PSI on the electron acceptor side, resulting in PSI photoinhibition under excess light conditions (Munekage et al., 2002). Despite the clear experimental evidence from mutant plants, the molecular mechanisms of PGR5 for P700 oxidation remain poorly understood and controversial. Since the protein was first identified, PGR5 has been proposed to drive CEF together with PGR5-like 1 protein (i.e., PGRL1) for the alleviation of the limitation of PSI on the electron acceptor side and for inducing lumen acidification (Munekage et al., 2002; Yamori and Shikanai, 2016; and references therein). Additionally, a lack of PGR5 impairs the association of FNR with the thylakoid membrane (Mosebach et al., 2017), indicating that PGR5 possibly affects photosynthetic linear electron flow (Takagi and Miyake, 2018). Furthermore, Kanazawa et al. (2017) suggests that PGR5 may function in adjusting the activity of ATPase rather than driving CEF, which is supported by the fact that the profiles of the mutants impaired in ATPase are strikingly similar to those of the PGR5 mutants (Kanazawa et al., 2017). Overall, the relationship of PGR5 with the photosynthetic electron transport system remains controversial.

The impact of PGR5 on P700 oxidation has changed from cyanobacteria to angiosperms. Although PGR5 is essential for P700 oxidation in angiosperms (Munekage et al., 2002), a lack of PGR5 has no effect on cyanobacterial photosynthesis (Allahverdiyeva et al., 2013). The contribution of PGR5 to P700 oxidation is observed in the green alga *C. reinhardtii* (Mosebach et al., 2017), indicating that a PGR5-dependent mechanism started to function as a P700 oxidation system after photosynthetic eukaryotes had evolved.

### Chloroplast NADPH Dehydrogenase (NDH)

Recently, it has been reported that NDH can also function as a P700 oxidation system under fluctuating light in C_3_ plant leaves. During a study using artificial, angularly incident, fluctuating light, mutants deficient in NDH showed impaired induction of P700 oxidation in *Arabidopsis thaliana* and *Oryza sativa* (Kono and Terashima, 2016; Yamori et al., 2016). The impact of NDH on P700 oxidation has also been tested during studies using sine-like artificial fluctuating light, named Umibozu, at different frequencies in *A. thaliana*, indicating that NDH is required for P700 oxidation only following a rapid change in light intensity under rapidly fluctuating light (Shimakawa and Miyake, 2018a). In chloroplasts of C_3_ plant leaves, NDH has effects on CEF (Shikanai et al., 1998) and chlororespiration (Sazanov et al., 1998). One hypothesis of the mechanism of NDH to support P700 oxidation is that NDH-dependent CEF functions under fluctuating light to produce ΔpH, limiting the electron transport in Cyt *b*_6_/*f* by photosynthetic control and accelerating the induction of photosynthesis to relieve the limitation of the electron acceptor side of PSI (Martin et al., 2015; Ishikawa et al., 2016; Kono and Terashima, 2016; Yamori et al., 2016). Other is that NDH contributes to oxidation of the chloroplast NADP^+^ pool in the darkness or low light in the process of chlororespiration, which can support the rapid start of P700 oxidation in response to the illumination with a fast fluctuating light (Shimakawa and Miyake, 2018a). Further studies are required on the detailed mechanisms of P700 oxidation by NDH.

In cyanobacteria, NDH can have a large effect on the redox state of both PQ and NADPH pools within cells, compared with that in plant leaves, because NDH also functions in the respiratory electron transport system (Mi et al., 2000; Ogawa et al., 2013). Similar to the case of terminal oxidases, it should be noted that the effects of NDH on P700 oxidation cannot be easily compared between cyanobacteria and photosynthetic eukaryotes.

## Rethinking the Mechanism of PSI Photoinhibition and the Dynamics of ROS in PSI

Recent studies have not only supported the hypothetical model of the mechanisms of PSI photoinhibition, established on the basis of the experimental findings of a study based on chilling stress (Sonoike, 2011), but have also provided novel insights into mechanisms for the generation of ROS in PSI. In this review, we revisited the hypothetical model of the mechanisms of PSI photoinhibition and the dynamics of ROS in PSI in oxygenic photoautotrophs. Originally, in chloroplasts the production of the ROS O_2_^-^ on the electron acceptor side of PSI has been defined as the Mehler reaction (Mehler, 1951). There are four electron acceptors for photo-excited P700 in PSI: chlorophyll A_0_ (primary acceptor), phylloquinone A_1_, and [4Fe–4S] clusters F_X_ and F_A_/F_B_. Both phylloquinones exist asymmetrically in the heterodimeric reaction centers of each of PsaA and PsaB (i.e., A_0A_, A_0B_, A_1A_, and A_1B_) (Joliot and Joliot, 1999). Among these acceptors, the Mehler reaction has been proposed to mainly occur at A_1B_ (Kruk et al., 2003) or F_X_ (Takahashi and Asada, 1988), which is supported by the lower midpoint redox potential (versus NHE) for A_1B_ (-820 mV) and F_X_ (-730 mV) (Brettel and Leibl, 2001; Kozuleva and Ivanov, 2010) than estimated (O_2_/O_2_^-^) in the lipid bilayer (from -500 to -600 mV versus NHE) (Wardman, 1990).

The increase in the photo-oxidative damage in PSI during rSP illumination in the range of light intensity for SP light, from 2000 to 20,000 μmol photons m^-2^ s^-1^ (Sejima et al., 2014), corresponds to the non-light saturation manner of the production of O_2_^-^ at phylloquinones in PSI (Kozuleva et al., 2014), which implies that O_2_^-^ produced by A_1B_ is likely to cause PSI photoinhibition. The production of O_2_^-^ can occur on both the stromal and luminal sides of PSI (Takahashi and Asada, 1988; Mubarakshina et al., 2006). O_2_^-^ produced during this process should immediately be disproportionated into H_2_O_2_ by the oxidation of ascorbate and superoxide dismutase in chloroplasts (Scarpa et al., 1983; Miyake and Asada, 1992). Further, H_2_O_2_ can react with the reduced [4Fe–4S] centers to produce ⋅OH where the acceptor side of PSI is reduced (Youngman and Elstner, 1981; Sonoike et al., 1997). The addition of methyl viologen, which strongly oxidizes the [4Fe–4S] centers and reduces O_2_ to O_2_^-^ on the stromal side of PSI, clearly alleviates PSI photoinhibition, which suggests that the photo-oxidative damage in PSI is caused by ⋅OH produced, depending on the reduced [4Fe–4S] centers (Sonoike, 1996) and/or ROS generated inside PSI (Takagi et al., 2016b).

Although all oxygenic photoautotrophs develop a variety of scavenging enzymes for ROS, including superoxide dismutase, ascorbate peroxidase, and catalase (Asada, 2006), at least in a physiological sense, these scavenging enzymes possibly have no impact on the alleviation of PSI photoinhibition. Originally, Terashima et al. (1998) has found the slight accumulation of H_2_O_2_ with the lower activity of thylakoid-bound ascorbate peroxidase in the C_3_ plant *Cucumis sativus* in the transition to chilling stress where PSI photoinhibition occurs in this plant. Whereas this finding clearly suggests that the ascorbate peroxidase modulates the H_2_O_2_ concentration in the plant leaves, it is still unclear if the accumulated H_2_O_2_ causes PSI photoinhibition. Overall, the effects of P700 oxidation can be lost in the isolated thylakoid membrane. Indeed, it has been proposed that the addition of superoxide dismutase and catalase do not alleviate PSI photoinhibition during a high light-stress treatment in an isolated PSI submembrane (Subramanyam et al., 2005). In the cyanobacterial FLV mutant that cannot keep P700 oxidized under CO_2_ limitation, PSI photoinhibition is rapidly induced even under constant light with approximately 200 μmol photons m^-2^ s^-1^ (Shimakawa et al., 2016b). In chloroplasts of plant leaves, PSI photoinhibition is observed during rSP illumination even in the presence of sufficient activities of superoxide dismutase and ascorbate peroxidase (Takagi et al., 2016b). These data clearly suggest that the scavenging enzymes of ROS cannot prevent PSI photoinhibition. That is, once produced, O_2_^-^ is supposed to immediately attack PSI and/or trigger the production of ⋅OH before it is scavenged, which may be supported by the significantly shorter lives of O_2_^-^ (2-4 μs) and ⋅OH (<1 μs) than that of H_2_O_2_ (1 ms; Van Breusegem et al., 2001). Contrarily, the production and diffusion of H_2_O_2_ can be easily detected in photosynthetic cells (Michelet et al., 2013; Roach et al., 2015), which is reasonable considering that H_2_O_2_ functions as a signaling molecule in oxygenic photoautotrophs (Van Breusegem et al., 2001; Gläßer et al., 2014; Dietz et al., 2016). Overall, we propose that the production of ROS leading to PSI photoinhibition can be completely distinguished from those related to dynamic metabolic and signaling mechanisms. It is possible that the different production site of O_2_^-^ causes the different effects of ROS on photosynthetic cells (Takagi et al., 2016b). Unfortunately, identification of the primary site in PSI attacked by ROS is still controversial (Tjus et al., 1999; Subramanyam et al., 2005; Takagi et al., 2016b), and more research is needed to identify the proper dynamics of ROS around PSI *in vivo*. The qualitative and quantitative relationships between the production of ROS and PSI photoinhibition should be addressed in future.

Besides O_2_^-^ and ⋅OH, singlet O_2_ (^1^O_2_) has been recently suggested to be generated in PSI and cause PSI photoinhibition. In the core and LHC complexes associated with PSI, triplet Chl can produce ^1^O_2_ to cause PSI photoinhibition, unless carotenoids such as β-carotene quench the triplet Chl (Subramanyam et al., 2005; Cazzaniga et al., 2012, 2016), which is considered a potential mechanism for alleviating PSI photoinhibition, in addition to P700 oxidation system. Long-lived triplet P700 suggests that ^1^O_2_ is unlikely to be generated from triplet P700 (Setif et al., 1981; Rutherford et al., 2012). Nevertheless, the generation of ^1^O_2_ originating from triplet P700 has recently been suggested during rSP illumination (Takagi et al., 2016b, 2017b). Ultimately, it is difficult to exclude the possibility that ^1^O_2_ has an impact on the photo-oxidative damage in PSI under severe excess light and in specific mutants.

## Concluding Remarks

Oxygenic photoautotrophs can safely undergo photosynthesis owing to P700 oxidation system. Despite the current poor understanding of the mechanisms of PSI photoinhibition, the effects of P700 oxidation on the alleviation of PSI photoinhibition discussed herein are likely to be true based on a number of experimental results (e.g., Figures 1–3). On the contrary, a recent study has reported an inconsistency between PSI photoinhibition and P700 oxidation in two different shade-established tropical tree species (Huang et al., 2015). Indeed, the degrees of PSI photoinhibition are diverse among a variety of oxygenic photoautotrophs, regardless of P700 oxidation levels (Takagi et al., 2017b), which probably reflects the different levels of robustness of PSI against ROS in each species. Most importantly, P700 oxidation is not directly linked to photosynthesis. Nevertheless, it is impossible for oxygenic photoautotrophs to live without P700 oxidation system because PSI photoinhibition is the lethal event for them (Shimakawa et al., 2016b). These facts reflect that all oxygenic photoautotrophs are confronted with the potential risk of photo-oxidative damage inevitably accompanied with exposure to light and O_2_. Diverse molecular mechanisms, i.e., P700 oxidation system, support P700 oxidation (Figure 4). On the contrary, almost all the agents of P700 oxidation system are still not characterized at the molecular level. There would be various ways to keep P700 oxidized. Intriguingly, the cyanobacterium *Leptolyngbya* sp., the species thriving in the harsh conditions of the desert, has been suggested to induce P700 oxidation by constricting the thylakoid lumen to limit diffusion of PC (Bar-Eyal et al., 2015). Unimaginable diversity of the strategies for P700 oxidation are possibly still unexplored in a variety of oxygenic photoautotrophs.

## Author Contributions

CM conceived the project and GS wrote the manuscript.

## Conflict of Interest Statement

The authors declare that the research was conducted in the absence of any commercial or financial relationships that could be construed as a potential conflict of interest. The reviewer YS declared a past co-authorship with the authors GS and CM to the handling Editor.
